# Correlation of anthropometric parameters with serum progesterone levels for the prediction of ovulation in infertile women in Ile‐Ife, Nigeria

**DOI:** 10.1002/ijgo.70959

**Published:** 2026-03-17

**Authors:** Akaninyene Eseme Ubom, Olumide Stephen Akinsomisoye, Muritala Abiola Asafa, Kelechi Nelson Adindu, Omobolanle Suliyat Ahmed, Tomiwa Akande

**Affiliations:** ^1^ Department of Physiological Sciences, Faculty of Basic Medical Sciences, College of Health Sciences Obafemi Awolowo University Ile‐Ife Nigeria; ^2^ Department of Obstetrics and Gynecology, Faculty of Clinical Sciences PAMO University of Medical Sciences Port Harcourt Nigeria; ^3^ School of Allied and Public Health University of Chester Chester UK

**Keywords:** anovulation, anthropometry, body mass index, cormic index, infertility, waist‐height ratio, waist‐hip ratio

## Abstract

**Objective:**

To correlate anthropometric parameters with serum levels of progesterone (P4) for the prediction of ovulation in infertile women.

**Methods:**

Body mass index (BMI), cormic index (CI), waist‐hip ratio (WHR), waist‐height ratio (WHtR), neck circumference (NC) and triceps skinfold thickness (TSF) were measured for 60 anovulatory and ovulatory infertile women, and fertile controls. Blood samples of the women were taken at the mid‐luteal phases of their menstrual cycles to determine their serum P4 levels. Pearson correlation coefficient was used to correlate the anthropometric parameters with serum P4 levels. Receiver operating characteristic (ROC) curve was utilized to determine critical anthropometry values to predict ovulation.

**Results:**

Mid‐luteal P4 had a significant moderate negative correlation with WHtR (*r* = −0.346, *P* = 0.008). Cormic index correlated significantly with WHtR (*r* = 0.265, *P* = 0.049). A CI of ≤43.6 was the best predictor of ovulation with an area under the curve (AUC) of 0.659 (95% CI: 0.511–0.806), with sensitivity, specificity, positive predictive value (PPV) and negative predictive value (NPV) of 78.8%, 57.1%, 75.7%, and 61.9%, respectively. A WHR of ≤0.83 was the second most accurate predictor of ovulation with an AUC of 0.559 (95% CI: 0.404–0.714), with sensitivity, specificity, PPV and NPV, respectively, of 77.8%, 31.8%, 65.1% and 46.7%.

**Conclusion:**

Cormic index could potentially be used alone or in combination with WHR and WHtR to predict ovulation amongst infertile women, obviating the need for routine costly hormonal assays. This would be especially useful in low‐resource settings with high levels of poverty, where insurance does not cover infertility investigations.

## 1 INTRODUCTION

Infertility affects more than 186 million individuals and approximately 48.5 million couples, representing 8%–12% of reproductive‐aged partners worldwide.^1^, ^2^ African women bear the highest burden of infertility globally. Infertility prevalence rates of between 9.5% and 32.0% have been reported in the region, with the majority of affected women living in the “infertility belt” of Central Africa, which extends from Tanzania to Gabon in the East and West, respectively.^2^, ^3^, ^4^ In Nigeria, a sub‐Saharan African country, there are more than 800 000 infertile couples, with infertility constituting more than one‐half of cases seen in gynecologic clinics in the country.^5^

Anovulation is the commonest cause of female factor infertility, accounting for 25% of infertility diagnoses,^6^ while tubal infertility is seen in 22% of infertile females.^7^ Male factor alone is implicated in 20%–30% of infertility diagnoses, and contributes to 50% of all infertility diagnoses.^2^, ^8^ To evaluate for ovulatory, tubal and male causes of infertility, three baseline infertility investigations are recommended, including evidence of ovulation, tubal patency test and semen analysis.^9^ According to the European Society of Human Reproduction and Embryology, these baseline infertility investigations cost an estimated €200–600, which is a significant expense in low‐resource settings.^10^ The American Society for Reproductive Medicine recommends that infertility evaluation should be conducted in a cost‐effective manner, with the initial emphasis on diagnostic modalities that are least invasive.^9^ Less invasive and cheap diagnostic modalities/prediction models save healthcare costs, especially in many low‐resource settings, where infertility investigations are either not routinely available, or are expensive and not covered by insurance.

Currently, the only clinical, non‐invasive and free‐cost adjunct that suggests and correlates positively with ovulation is regularity of menstrual cycles.^11^ However, ascertaining the regularity of a woman's menstrual cycle is prone to errors as it depends on the woman's memory and her understanding of a regular menstrual cycle. Anthropometric parameters, including elevated body mass index (BMI, calculated as weight in kilograms divided by height in meters squared) and elevated waist‐hip ratio (WHR), have been associated with anovulation.^12^, ^13^ However, there are no established critical BMI or WHR values to clinically predict ovulation. Literature is also very sparse on the relationship of other anthropometric parameters, including cormic index (CI), waist‐height ratio (WHtR), neck circumference (NC) and triceps skin fold thickness (TSF), with ovulation. The present study aimed to correlate the anthropometric parameters of BMI, CI, WHtR, WHR, NC and TSF, with the reference standard of serum levels of progesterone (P4) to determine critical values of these anthropometric parameters that could predict ovulation in infertile women.

## 2 MATERIALS AND METHODS

### 2.1 Study design, population and setting

The present case–control study was conducted amongst reproductive age women attending Gynecology Clinics at the Obafemi Awolowo University Teaching Hospitals Complex (OAUTHC), Ile‐Ife, Nigeria.

### 2.2 Inclusion and exclusion criteria

Consenting women with infertility, aged 18–49 years, were included as cases; while consenting fertile women were recruited as controls. Women with a history of non‐steroidal anti‐inflammatory drug use for mid‐cycle pain, pregnant and lactating women, were excluded.

### 2.3 Sample size determination

The sample size for this study was determined using the sample size formula for case–control studies^14^:

$$N=\left\lfloor \frac{2\times {\left(Z_{\alpha }+Z_{\beta }\right)}^{2}\times P\times \left(1-P\right)}{{\left(P_{0}-P_{1}\right)}^{2}}\right\rfloor$$

where: *N* = sample size; *Z*_α_ = standard normal deviation for a two‐tailed alpha error of 0.05 = 1.96; *Z*_𝛽_ = standard normal deviation for a power of 80% = 0.84; *P*_0_ = proportion of women with infertility expected to be anovulatory = 0.25 (25%)^7^; *P*_1_ = proportion of women with infertility expected to be ovulatory = 0.75 (75%). Substituting these values into the sample size formula gave an approximate sample size of 16. Factoring in a 10% attrition rate brought the sample size to approximately 18, rounded off to 20. The women were, therefore, divided into three equal groups of 20 women each: Group I—anovulatory infertile women, group II—ovulatory infertile women, and group III—fertile controls. Group I included infertile women with mid‐luteal serum P4 value of <10 ng/mL (<30 nmol/L); group II included infertile women with mid‐luteal serum P4 value of ≥10 ng/mL (≥30 nmol/L); and group III included fertile women with mid‐luteal serum P4 value of ≥10 ng/mL (≥30 nmol/L).

### 2.4 Study procedure

The baseline characteristics of the study participants, including age, parity, type, duration and cause of infertility, were obtained and documented in a purpose‐designed proforma.

### 2.5 Anthropometric measurements

The following anthropometric measurements were taken and recorded for all study participants:

- *Standing and sitting heights*: The standing heights of the study participants were measured with a stadiometer (ZT‐160 Surgilac, Wincom Company Ltd., China) and recorded in meters (m). To measure sitting height, the women were made to sit on a chair placed against the vertical measuring stand of a stadiometer (ZT‐160 Surgilac, Wincom Company Ltd., China), and the total height from the top of the woman's head to the floor was measured. The height of the chair was subtracted from this total height to obtain the sitting height.
- *Weight*: The weights of the study participants were measured using the analog weighing scale of the stadiometer (ZT‐160 Surgilac, Wincom Company Ltd., China) and recorded in kilograms (kg).
- *Waist circumference* (WC): This was measured using a medical tape measure (SKUSLD00035, Medshop, Australia) and recorded in cm. The measurement was taken at the narrowest part of the torso, just above the umbilicus, mid‐distance between the inferior margin of the ribs and the superior border of the iliac crests.
- *Hip circumference* (HC): The HC was measured around the widest part of the buttocks, at the level of the greater trochanters, with a medical tape measure (SKUSLD00035, Medshop, Australia) and recorded in centimeters (cm).
- *Neck circumference* (NC): The NC was measured by wrapping a medical tape measure (SKUSLD00035, Medshop, Australia) around the neck, just below the larynx (Adam's apple), and recorded in cm.
- *Triceps skinfold thickness* (TSF): This was measured over the triceps at the mid‐portion of the posterior upper arm, located mid‐distance between the acromion process of the humerus and the olecranon process, by firmly grasping the skin fold between the index finger and thumb, taking care not to include underlying muscle. The skin fold thickness was measured with a skinfold caliper (Lange, Beta Technology Inc., USA) and recorded in cm.
- *Body mass index* (BMI): The BMI was calculated using the formula: BMI = (weight in kg/[height in m^2^]) and recorded in kg/m^2^.
- *Cormic index* (CI): The CI was calculated with the formula (sitting height/standing height) × 100.
- *Waist‐hip ratio* (WHR): This was determined by dividing the WC by the HC.
- *Waist‐height ratio* (WHtR): This was determined by dividing the WC by the height.

### 2.6 Hormonal assay

At the mid‐luteal phase, 5 mL of venous blood was obtained from all the study participants and analyzed in the Chemical Pathology Laboratory of the OAUTHC, to determine the P4 level. The blood samples were collected in a universal plain bottle, transported to the laboratory within 1 h, and allowed to clot adequately. Serum was obtained after retraction by centrifuging at 3000 revolutions per minute for 5 min. The separated serum was kept frozen at −80°C for later analyses. Serum levels of P4 in the blood samples were determined using the iChroma autoanalyzer (Boditech Med Inc., Republic of Korea), a fully automated analyzer that utilizes fluorescence immunoassay (FIA) combined with a sandwich immunodetection method to quantitatively determine P4 levels in human serum: the fluorescence‐labeled detector antibody in the detection buffer binds to antigen/target material in a sample, forming antigen–antibody complexes. These complexes when loaded into cartridges containing test strips migrate onto the nitrocellulose matrix and the antigen/target material is immobilized on the test strips.

To perform the procedure, using a transfer pipette, 150 μL of the human serum sample was transferred to the detection buffer in a tube. The lid of the buffer tube was closed and the sample was thoroughly mixed by shaking it approximately 10 times. Thereafter, a pipette was used to remove 75 μL of the sample mixture, which was loaded into the sample well on the test cartridge. The sample‐loaded test cartridge was allowed at room temperature (25°C) for 15 min. To scan the sample‐loaded cartridge, it was inserted into the cartridge holder of the iChroma machine and the “Select” button on the machine was pressed. The serum level of P4 was then read on the display screen of the machine. A mid‐luteal serum P4 value of ≥10 ng/mL (≥30 nmol/L) was considered confirmatory of ovulation.^15^, ^16^, ^17^

### 2.7 Reliability criteria for P4 assay

The following reliability criteria for the P4 assay were ensured.

#### 2.7.1 Accuracy

Assay accuracy was ensured by simultaneous analysis of commercially prepared control sample alongside the study participants' samples. This parallel analysis helped to validate the accuracy of the iChroma immunoassay results.

#### 2.7.2 Precision

Each study participant's serum P4 level was measured once; there were no duplicate or repeated assays to allow for assessment of assay precision. However, the use of a fully automated iChroma analyzer inherently improved precision by minimizing manual variations as the equipment uses a consistent and standardized automated protocol for all test sample assays. The Boditech Med iChroma has a low margin of error of 5.2%–6.6%.

#### 2.7.3 Analytical sensitivity

The Boditech Med iChroma autoanalyzer used in this study utilizes FIA to quantitatively measure serum P4 levels, with an analytical sensitivity range of 1.4–40 ng/mL (4.45–127.2 nmol/L), making it able to detect even very low hormone levels.

#### 2.7.4 Analytical specificity

The iChroma P4 test is highly specific for P4 assay as it utilizes the sandwich immunodetection method with hormone‐specific antibodies. The fluorescence‐labeled antibody binds specifically to P4 in the serum sample, forming an antigen–antibody complex that is subsequently detected on a test strip. This method ensures that the hormonal assay is P4 specific, thereby limiting cross‐reactivity with other hormones. The study relied on the established design of the iChroma autoanalyzer to ensure analytical specificity, and no off‐target binding issues were identified during the study.

#### 2.7.5 Intra‐ and inter‐assay variability

This study minimized variability by ensuring that all blood samples were processed under consistent conditions. Inter‐assay variability was minimized by freezing samples and analyzing them in the same laboratory with the same equipment and under similar conditions for each batch of test samples. Intra‐assay variability was mitigated by the automated design of the iChroma analyzer, as it performs the assay steps uniformly for each sample.

#### 2.7.6 Reliability

Reliability was ensured by strict adherence to the same assay protocol/procedure for all test samples.

### 2.8 Statistical analysis

Data obtained from this study were analyzed using the IBM SPSS Statistics version 25. Correlation of anthropometric parameters with serum levels of P4 was carried out using Pearson correlation coefficient. A Pearson correlation coefficient of 0.10–0.29 indicated a weak correlation, 0.30–0.49, moderate correlation, and 0.50–1.0, strong correlation.^18^

Receiver operating characteristic (ROC) curves were used to determine the optimal predictive values, sensitivity, specificity, negative and positive predictive values of BMI, CI, WHR, WHtR, TSF, and NC. The cutoffs of 90%–100%, >80%–<90%, 65%–80%, and <65% indicated very high, high, moderate and low sensitivity and specificity, respectively.^19^ An area under the curve (AUC) value of 1.0 indicated a perfect test (no false positives or negatives), 0.9–0.99 an excellent test, 0.8–0.89 a good test, 0.7–0.79 a fair test and <0.7 a non‐useful test.^20^ Statistical significance was determined at a *P* value less than 0.05.

Model quality scores were determined for each of the anthropometric parameters to assess their accuracy in predicting ovulation. The model quality scores summarized how well the various anthropometric parameters performed in predicting ovulation by combining sensitivity and specificity into a single value. A good model has a model quality score above 0.5, while a value less than 0.5 indicates that the model is no better than random prediction.^21^

### 2.9 Ethics

The Research and Ethics Committee of the OAUTHC granted ethical approval for this study (ERC/2024/04/11). Informed consents for study participation were obtained from all the study participants prior to recruitment for the study.

## 3 RESULTS

### 3.1 Gynecologic, anthropometric, and hormonal characteristics of the study participants and comparisons between groups

Table 1 shows the baseline gynecologic, anthropometry and hormonal parameters of the study participants. The mean BMIs of women in the three groups were all in the overweight range. The mean BMI (28.88 ± 6.43 kg/m^2^ vs. 25.31 ± 6.36 kg/m^2^, *P* = 0.086), CI (43.48 ± 2.92 vs. 44.61 ± 3.93, *P* = 0.327), WHR (0.86 ± 0.07 vs. 0.88 ± 0.08, *P* = 0.424), WHtR (0.57 ± 0.07 vs. 0.54 ± 0.08, *P* = 0.268), NC (34.95 ± 3.00 cm vs. 32.83 ± 5.66 cm, *P* = 0.147), and TSF (7.70 ± 2.29 cm vs. 9.00 ± 3.00 cm, *P* = 0.136), were comparable between anovulatory (group I) and ovulatory (group II) infertile women (Tables 1 and 2). Day 21 serum P4 level was significantly higher amongst women in group II than women in group I (18.42 ng/mL vs. 2.20 ng/mL, *P* < 0.001) (Tables 1 and 2).

**TABLE 1:** Baseline characteristics of the study participants.

Characteristics	Frequency (%)		
	Group I (anovulatory infertile women), *n =* 20	Group II (ovulatory infertile women), *n =* 20	Group III (fertile controls), *n =* 20
Mean age ± SD (years)	35.35 ± 5.43	34.80 ± 6.01	37.00 ± 6.22
Median parity (IQR)	0 (0–1)	0 (0–1)	2 (1–3)
Type of infertility			
Primary	6 (30.0)	10 (50.0)	—
Secondary	14 (70.0)	10 (50.0)	—
Cause of infertility			
Polycystic ovary syndrome	16 (80.0)	0 (0.0)	—
Hyperprolactinemia	4 (20.0)	0 (0.0)	—
Male factor	7 (35.0)	11 (55.0)	—
Tubal factor	1 (5.0)	7 (35.0)	—
Uterine factor	1 (5.0)	4 (20.0)	—
Unexplained	0 (0.0)	3 (15.0)	
Median (IQR) duration of infertility (months)	36 (24–72)	48 (18–84)	—
Mean ± SD BMI (kg/m^2^)	28.88 ± 6.43	25.31 ± 6.36	25.50 ± 5.60
Mean ± SD CI	43.48 ± 2.92	44.61 ± 3.93	46.43 ± 2.16
Mean ± SD WHR	0.86 ± 0.07	0.88 ± 0.08	0.85 ± 0.06
Mean ± SD WHtR	0.57 ± 0.07	0.54 ± 0.08	0.53 ± 0.08
Mean ± SD NC (cm)	34.95 ± 3.00	32.83 ± 5.66	34.25 ± 2.94
Mean ± SD TSF (cm)	7.70 ± 2.29	9.00 ± 3.00	7.20 ± 3.21
Median (IQR) Day 21 P4 (ng/mL)	2.20 (0.23–3.90)	18.42 ± 5.23	22.25 (15.70–28.35)

*Note*: BMI, calculated as weight in kilograms divided by height in meters squared. *n* > 20 for Cause of infertility due to multiple combined causes in some patients.

Abbreviations: BMI, body mass index; CI, cormic index; IQR, interquartile range; NC, neck circumference; SD, standard deviation; TSF, triceps skin fold thickness; WHR, waist‐hip ratio; WHtR, waist‐height ratio.

**TABLE 2:** Comparison of gynecologic, anthropometric and hormonal parameters between women in groups I (anovulatory infertile women) and II (ovulatory infertile women).

Characteristic	Group I (*n =* 20)	Group II (*n =* 20)	*P* value
Mean ± SD age (years)	35.35 ± 5.43	34.80 ± 6.01	0.763^a^
Median (IQR) parity	0 (0–1)	0 (0–1)	0.370^b^
Mean ± SD BMI (kg/m^2^)	28.88 ± 6.43	25.31 ± 6.36	0.086^a^
Mean ± SD CI	43.48 ± 2.92	44.61 ± 3.93	0.327^a^
Mean ± SD WHR	0.86 ± 0.07	0.88 ± 0.08	0.424^a^
Mean ± SD WHtR	0.57 ± 0.07	0.54 ± 0.08	0.268^a^
Mean ± SD NC (cm)	34.95 ± 3.00	32.83 ± 5.66	0.147^a^
Mean ± SD TSF (cm)	7.70 ± 2.29	9.00 ± 3.00	0.136^a^
Day 21 P4 level (ng/mL)	2.20 (0.23–3.90)	18.42 ± 5.23	<0.001^b^ ^,^ *

*Note*: BMI, calculated as weight in kilograms divided by height in meters squared. Mean ± SD for normally distributed data and median (IQR) for skewed data.

Abbreviations: BMI, body mass index; CI, cormic index; IQR, interquartile range; NC, neck circumference; SD, standard deviation; TSF, triceps skin fold thickness; WHR, waist‐hip ratio; WHtR, waist‐height ratio.

^a^ Student *t*‐test.

^b^ Mann–Whitney *U* test.

* Statistical significance.

The mean BMI (28.88 ± 6.43 kg/m^2^ vs. 25.50 ± 5.60 kg/m^2^, *P* = 0.085), WHR (0.86 ± 0.07 vs. 0.85 ± 0.06, *P* = 0.650), WHtR (0.57 ± 0.07 vs. 0.53 ± 0.08, *P* = 0.166), NC (34.95 ± 3.00 cm vs. 34.25 ± 2.94 cm, *P* = 0.461) and TSF (7.70 ± 2.29 cm vs. 7.20 ± 3.21 cm, *P* = 0.574) were higher in anovulatory infertile (group I) women compared to fertile controls (group III), though the differences were not statistically significant (Tables 1 and 3). The mean CI (46.43 ± 2.16 vs. 43.48 ± 2.92, *P* = 0.001) and median day 21 serum P4 level (22.25 ng/mL vs. 2.20 ng/mL, *P* < 0.001) were significantly higher amongst women in group III than those in group I (Tables 1 and 3).

**TABLE 3:** Comparison of gynecologic, anthropometric, and hormonal parameters between women in groups I (anovulatory infertile women) and III (fertile controls).

Characteristic	Group I (*n =* 20)	Group III (*n =* 20)	*P* value
Mean ± SD age (years)	35.35 ± 5.43	37.00 ± 6.22	0.377^a^
Median (IQR) parity	0 (0–1)	2 (1–3)	<0.001^b^ ^,^ *
Mean ± SD BMI (kg/m^2^)	28.88 ± 6.43	25.50 ± 5.60	0.085^a^
Mean ± SD CI	43.48 ± 2.92	46.43 ± 2.16	0.001^a^ ^,^ *
Mean ± SD WHR	0.86 ± 0.07	0.85 ± 0.06	0.650^a^
Mean ± SD WHtR	0.57 ± 0.07	0.53 ± 0.08	0.166^a^
Mean ± SD NC (cm)	34.95 ± 3.00	34.25 ± 2.94	0.461^a^
Mean ± SD TSF (cm)	7.70 ± 2.29	7.20 ± 3.21	0.574^a^
Day 21 P4 level (ng/mL)	2.20 (0.23–3.90)	22.25 (15.70–28.35)	<0.001^b^ ^,^ *

*Note*: BMI, calculated as weight in kilograms divided by height in meters squared. Mean ± SD for normally distributed data and median (IQR) for skewed data.

Abbreviations: BMI, body mass index; CI, cormic index; IQR, interquartile range; NC, neck circumference; SD, standard deviation; TSF, triceps skin fold thickness; WHR, waist‐hip ratio; WHtR, waist‐height ratio.

^a^ Student *t*‐test.

^b^ Mann–Whitney *U* test.

* Statistical significance (*P* < 0.05).

Comparing ovulatory infertile (group II) women and fertile controls (group III), mean BMI (25.31 ± 6.36 kg/m^2^ vs. 25.50 ± 5.60 kg/m^2^, *P* = 0.917), CI (44.61 ± 3.93 vs. 46.43 ± 2.16, *P* = 0.086), WHR (0.88 ± 0.08 vs. 0.85 ± 0.06, *P* = 0.201), WHtR (0.54 ± 0.08 vs. 0.53 ± 0.08, *P* = 0.814), NC (32.83 ± 5.66 cm vs. 34.25 ± 2.94 cm, *P* = 0.324), and TSF (9.00 ± 3.00 cm vs. 7.20 ± 3.21 cm, *P* = 0.079), and serum P4 levels (18.42 ng/mL vs. 22.25 ng/mL, *P* = 0.236) were comparable between the two groups (Tables 1 and 4).

**TABLE 4:** Comparison of gynecologic, anthropometric, and hormonal parameters between women in groups II (ovulatory infertile women) and III (fertile controls).

Characteristic	Group II (*n =* 20)	Group III (*n =* 20)	*P* value
Mean ± SD age (years)	34.80 ± 6.01	37.00 ± 6.22	0.262^a^
Median (IQR) parity	0 (0–1)	2 (1–3)	<0.001^b^ ^,^ *
Mean ± SD BMI (kg/m^2^)	25.31 ± 6.36	25.50 ± 5.60	0.917^a^
Mean ± SD CI	44.61 ± 3.93	46.43 ± 2.16	0.086^a^
Mean ± SD WHR	0.88 ± 0.08	0.85 ± 0.06	0.201^a^
Mean ± SD WHtR	0.54 ± 0.08	0.53 ± 0.08	0.814^a^
Mean ± SD NC (cm)	32.83 ± 5.66	34.25 ± 2.94	0.324^a^
Mean ± SD TSF (cm)	9.00 ± 3.00	7.20 ± 3.21	0.079^a^
Day 21 P4 level (ng/mL)	18.42 ± 5.23	22.25 (15.70–28.35)	0.236^b^

*Note*: BMI, calculated as weight in kilograms divided by height in meters squared. Mean ± SD for normally distributed data and median (IQR) for skewed data.

Abbreviations: BMI, body mass index; CI, cormic index; IQR, interquartile range; NC, neck circumference; SD, standard deviation; TSF; triceps skin fold thickness; WHR, waist‐hip ratio; WHtR, waist‐height ratio.

^a^ Student *t*‐test.

^b^ Mann–Whitney *U* test.

* Statistical significance (*P* < 0.05).

### 3.2 Correlation of anthropometric parameters with mid‐luteal P4 levels

Pearson correlation indicated that there was significant moderate negative correlation between BMI (*r* = −0.434, *P* = 0.001) and WHtR (*r* = −0.346, *P* = 0.008) and mid‐luteal serum P4. There was a weak positive correlation between CI (*r* = 0.186, *P* = 0.178) and mid‐luteal serum P4, while WHR (*r* = −0.009, *P* = 0.946), NC (*r* = −0.241, *P* = 0.068) and TSF (*r* = −0.060, *P* = 0.657) had weak negative correlation with mid‐luteal P4 (Table 5). Cormic index significantly correlated with WHtR (*r* = 0.265, *P* = 0.049), but not with BMI (*r* = 0.223, *P* = 0.098) and WHR (*r* = 0.144, *P* = 0.290).

**TABLE 5:** Correlation of anthropometric parameters with mid‐luteal P4 levels.

Anthropometry	Pearson correlation coefficient, *r*	*P* value	Interpretation
BMI	−0.434	0.001*	Significant moderate negative correlation
CI	0.186	0.178	Weak positive correlation
WHR	−0.009	0.946	Weak negative correlation
WHtR	−0.346	0.008*	Significant moderate negative correlation
NC	−0.241	0.068	Weak negative correlation
TSF	−0.060	0.657	Weak negative correlation

*Note*: BMI, calculated as weight in kilograms divided by height in meters squared.

Abbreviations: BMI, body mass index; CI, cormic index; NC, neck circumference; TSF; triceps skin fold thickness; WHR, waist‐hip ratio; WHtR, waist‐height ratio.

* Statistical significance (*P* < 0.05).

### 3.3 Critical values of anthropometric parameters for the prediction of ovulation

**FIGURE 1:**
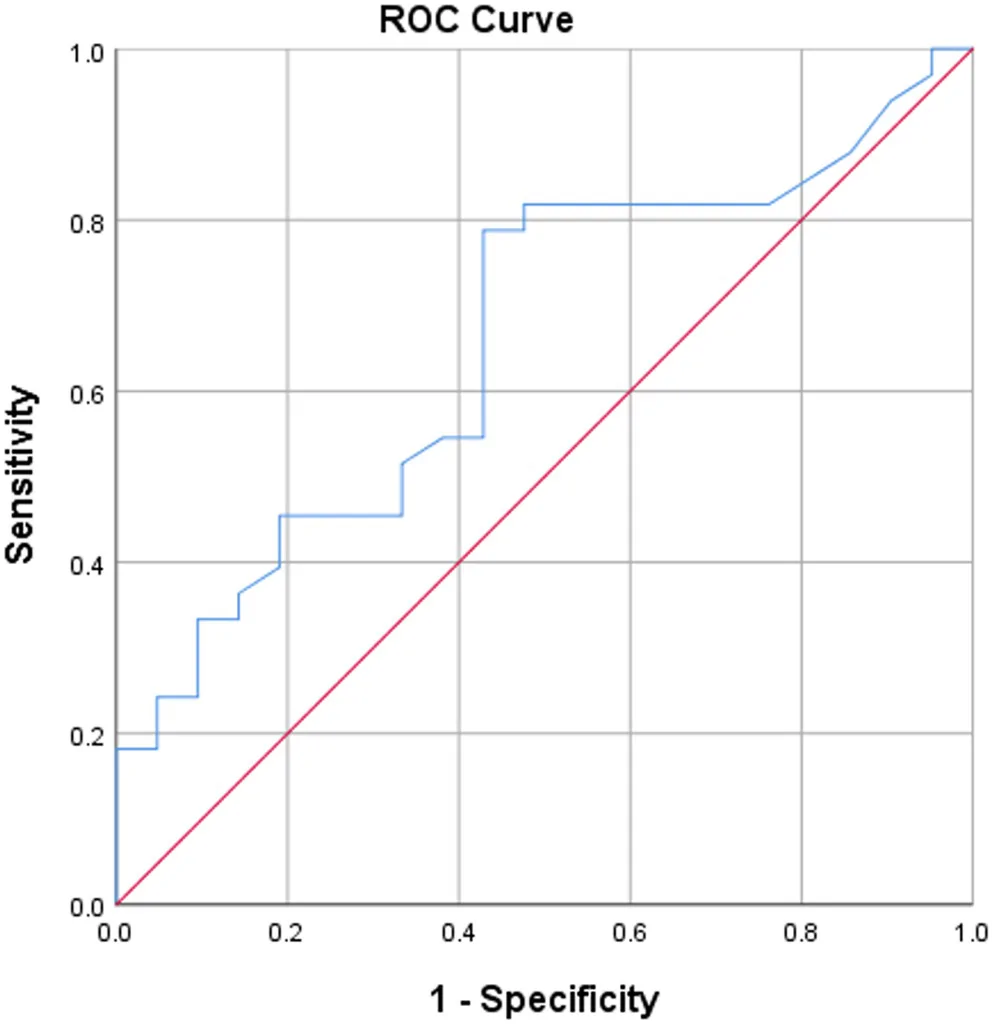
Receiver operating characteristic (ROC) curve for various cutoff values of cormic index (CI) for predicting ovulation.

**FIGURE 2:**
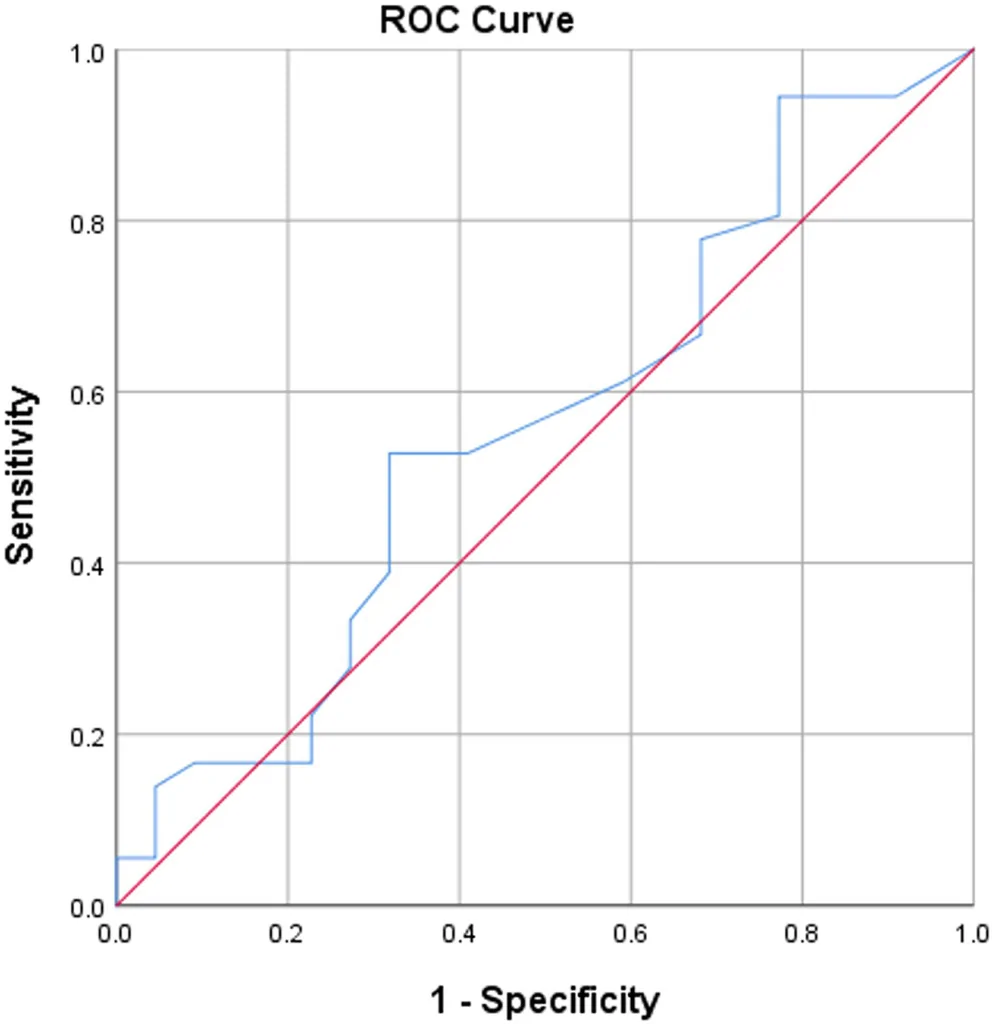
Receiver operating characteristic (ROC) curve for various cutoff values of waist‐hip ratio (WHR) for predicting ovulation.

Table 6 shows the critical cutoff values of BMI, CI, WHR, WHtR, NC and TSF, which were predictive of ovulation, with sensitivity, specificity, negative predictive value (NPV) and positive predictive value (PPV) for each cutoff. Cormic index was the most accurate predictor of ovulation as seen in the ROC curve in Figure 1, with an AUC of 0.659 (95% CI: 0.511–0.806). A CI cutoff of ≤43.6 had a 78.8% sensitivity and 57.1% specificity for predicting ovulation, with a PPV of 75.7%, and NPV of 61.9%. Waist‐hip ratio was the second most accurate predictor of ovulation, with an AUC of 0.559 (95% CI: 0.404–0.714) (Figure 2). A WHR cutoff of ≤0.83 had a sensitivity of 77.8%, specificity of 31.8%, PPV of 65.1% and NPV of 46.7%, for the prediction of ovulation.

**TABLE 6:** Critical values of anthropometric parameters for predicting ovulation.

Parameter	AUC (95% CI)	Optimal cutoff	Sensitivity (%)	Specificity (%)	PPV (%)	NPV (%)
CI	0.659 (0.511–0.806)	≤43.60	78.8	57.1	75.7	61.9
WHR	0.559 (0.404–0.714)	≤0.83	77.8	31.8	65.1	46.7
TSF (cm)	0.533 (0.382–0.684)	≤6.80	71.4	36.4	65.0	44.4
NC (cm)	0.383 (0.229–0.538)	≤32.30	66.7	27.3	60.0	33.3
WHtR	0.372 (0.224–0.520)	≤0.49	66.7	22.7	58.5	29.4
BMI (kg/m^2^)	0.326 (0.186–0.467)	≤24.0	52.8	31.8	55.9	29.2

*Note*: BMI, calculated as weight in kilograms divided by height in meters squared.

Abbreviations: AUC, area under the curve; BMI, body mass index; CI, cormic index; NC, neck circumference; NPV, negative predictive value; PPV, positive predictive value: TSF; triceps skin fold thickness; WHR, waist‐hip ratio; WHtR, waist‐height ratio.

**FIGURE 3:**
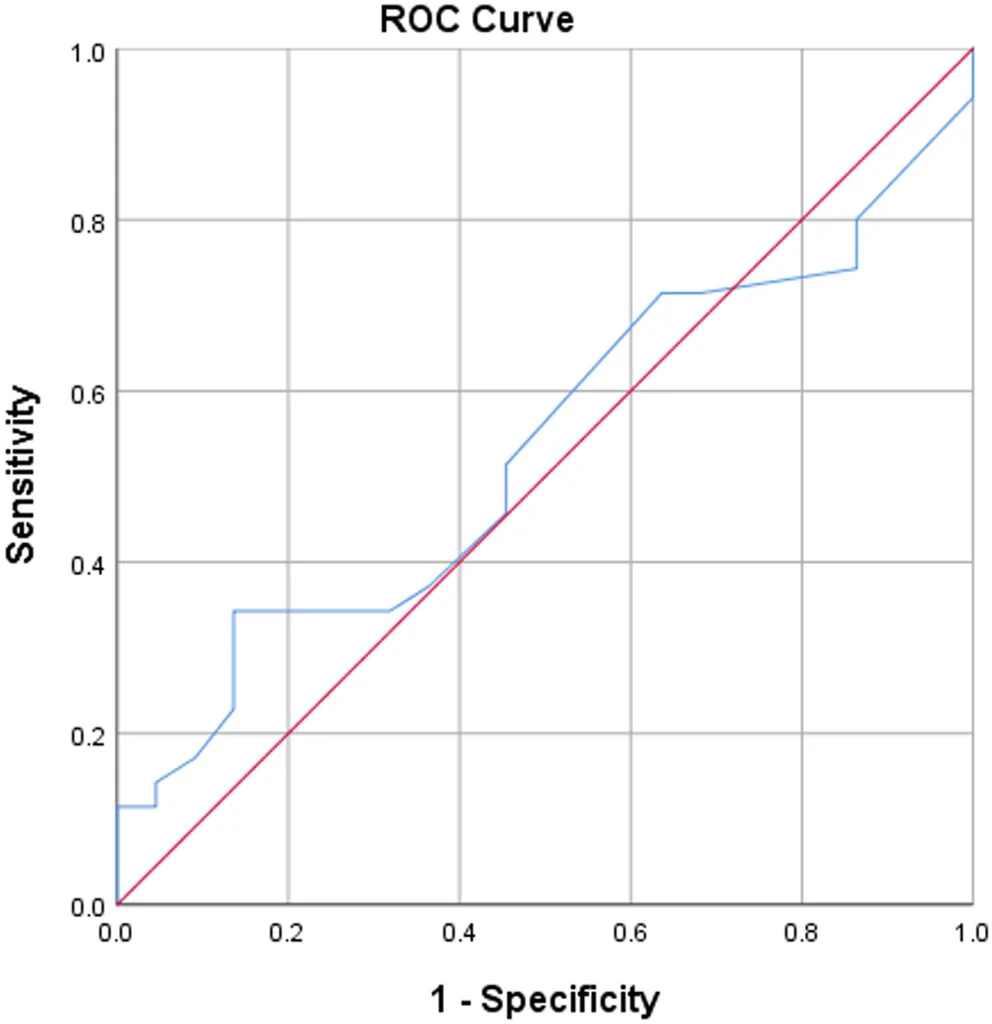
Receiver operating characteristic (ROC) curve for various cutoff values of triceps skin fold thickness (TSF) for predicting ovulation.

**FIGURE 4:**
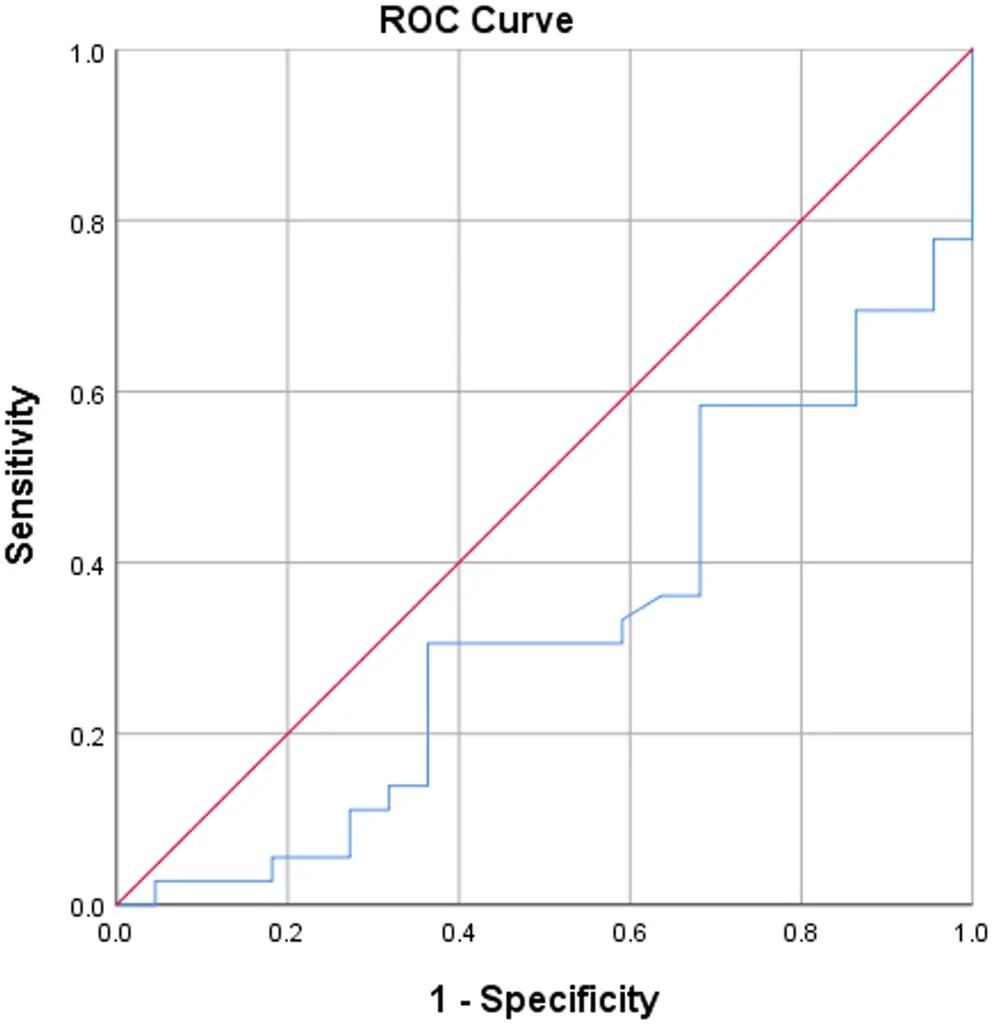
Receiver operating characteristic (ROC) curve for various cutoff values of body mass index (BMI) for predicting ovulation.

**FIGURE 5:**
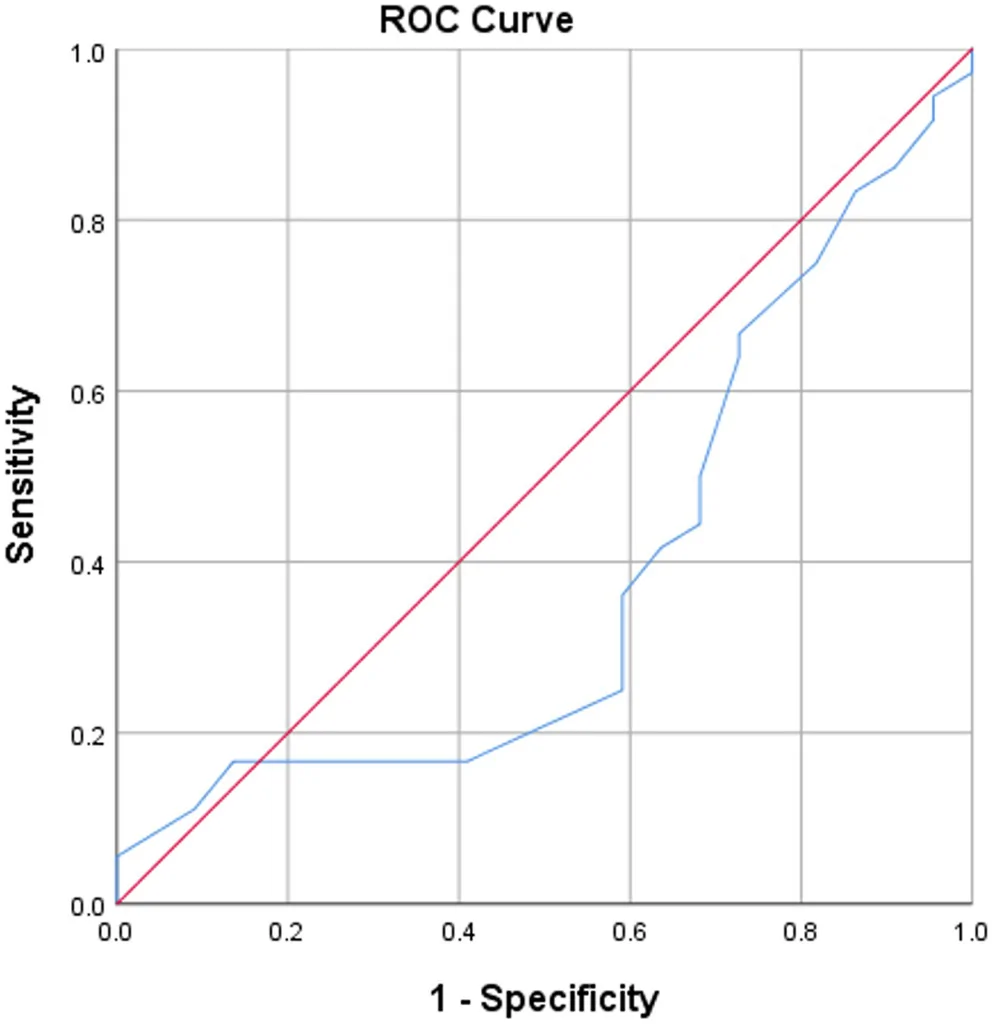
Receiver operating characteristic (ROC) curve for various cutoff values of neck circumference (NC) for predicting ovulation.

**FIGURE 6:**
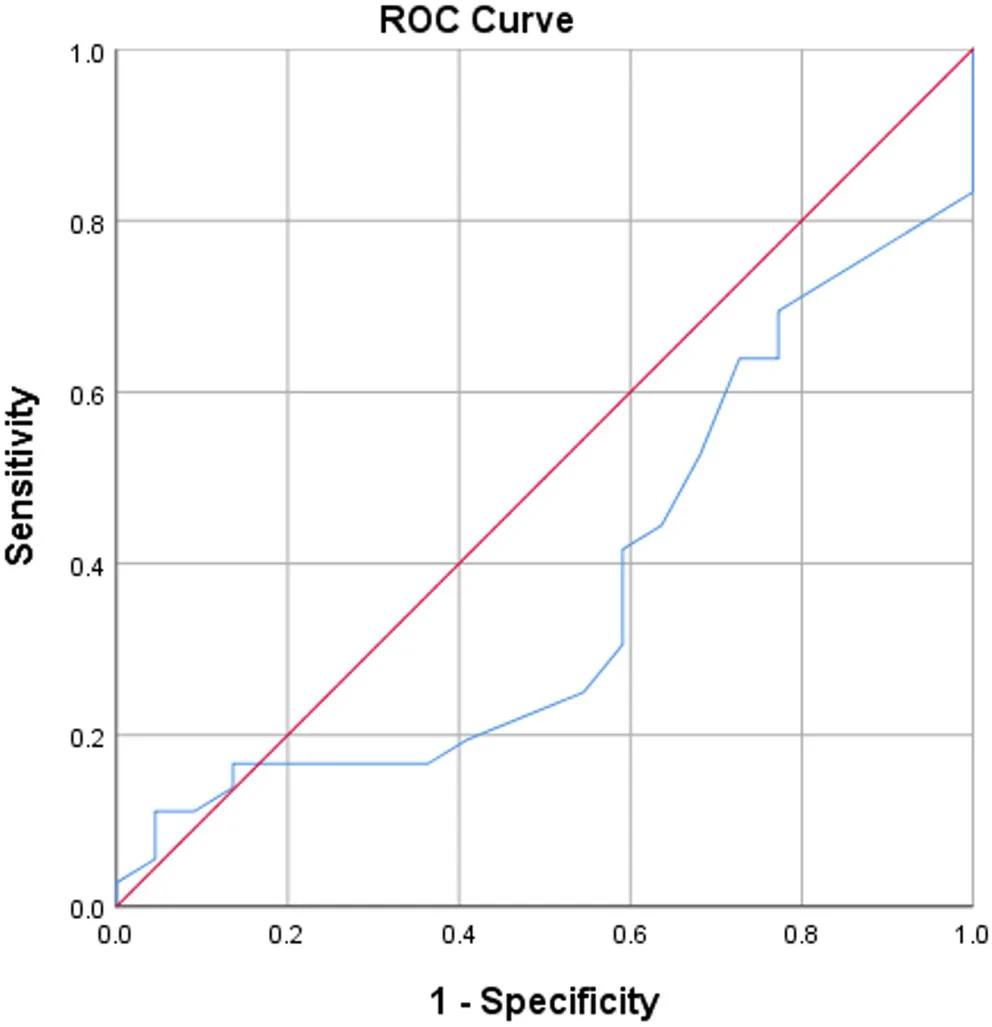
Receiver operating characteristic (ROC) curve for various cutoff values of waist‐height ratio (WHtR) for predicting ovulation.

After CI and WHR, TSF was the third most reliable predictor of ovulation, with an AUC of 0.533 (95% CI: 0.382–0.684) (Figure 3). BMI was a poor predictor of ovulation, with an AUC of 0.326 (95% CI: 0.186–0.467) (Figure 4). Neck circumference and WHtR were also poor, but better predictors of ovulation than BMI, with AUCs of 0.383 (95% CI: 0.229–0.538) and 0.372 (95% CI: 0.224–0.520), respectively (Figures 5 and 6).

### 3.4 Model quality scores of anthropometric parameters for the prediction of ovulation

**FIGURE 7:**
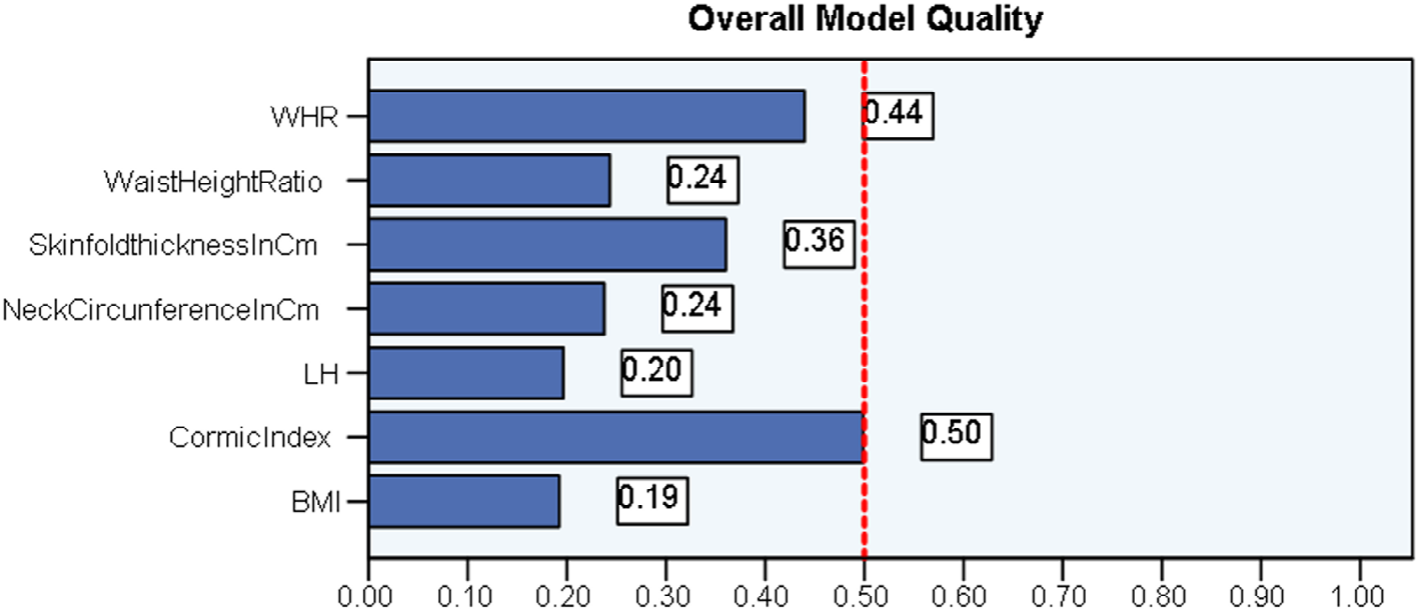
Model quality scores of body mass index (BMI), cormic index (CI), waist‐hip ratio (WHR), waist‐height ratio (WHtR), neck circumference (NC), triceps skin fold thickness (TSF) and luteinizing hormone (LH) for the prediction of ovulation.

Cormic index had the highest overall model quality score of 0.5 for the prediction of ovulation. The overall model quality scores of all the other anthropometric parameters were <0.5, at 0.44, 0.36, 0.24, 0.24, 0.19 for WHR, TSF, NC, WHtR and BMI, respectively (Figure 7).

## 4 DISCUSSION

Of the six anthropometric parameters assessed in this study, CI was the most accurate predictor of ovulation, with an AUC of 0.7, and moderate sensitivity and PPV of 78.8% and 75.7%, respectively. Waist‐hip ratio was the second most accurate predictor of ovulation, with an AUC of 0.6, moderate sensitivity of 77.8%, and PPV of 65.1%. Within the limits of available literature search, the association between CI and ovulation noted in this study has not been previously reported. Furthermore, CI had a significant positive correlation with WHtR, while WHtR had a significant moderate negative correlation with mid‐luteal P4 level in this study. Waist‐height ratio was higher in anovulatory infertile women compared to both ovulatory infertile women and fertile controls. Similarly, anovulatory infertile women had a slightly higher WHR than their fertile counterparts. Whereas BMI measures general obesity, WHR and WHtR are specific for abdominal obesity, a significant risk factor for insulin resistance and anovulation.^22^, ^23^ Waist‐hip ratio and WHtR are, therefore, considered superior to BMI for predicting central adiposity and anovulation.^24^, ^25^

In this study, aside from sensitivity, specificity, PPV and NPV, model quality scores were determined for all the anthropometric parameters to measure how well they performed in predicting ovulation. Reporting individual performance metrics such as sensitivity, specificity, PPV, NPV and AUC, does not adequately highlight the predictive value of models; an overall model quality score summarizes the global predictive strength of a model. Cormic index had the highest overall model quality score of 0.5 for the prediction of ovulation in this study; the overall model quality scores of all the other anthropometric parameters were <0.5—WHR performed better than the other anthropometric parameters, with a model quality score of 0.44.

The significant correlation of WHtR with both P4 and CI, and the highest sensitivity and overall model quality scores of CI and WHR for the prediction of ovulation in this study, underscore the potential of CI being used either alone, or in combination with WHR and WHtR, for the prediction of ovulation. This potential has to be further assessed with highly powered, larger studies, as some authors have recommended that a minimum sample of 300 produces the highest accuracy for evaluating the sensitivity and specificity of a screening test/tool for population‐wide use.^26^, ^27^ A limitation of using anthropometry to predict ovulation, which may have affected this study findings, and which should be considered and corrected for in further studies, is the fact that anthropometry is affected by many factors including genetics, heredity, environmental factors, age, geography, race, socioeconomic factors and nutritional status, amongst others.^28^, ^29^

## AUTHOR CONTRIBUTIONS

**Akaninyene Eseme Ubom**: Conceptualization (lead); investigation (lead); methodology (lead); resources (lead); formal analysis (lead); visualization (lead); writing—original draft (lead); writing—review and editing (equal). **Olumide Stephen Akinsomisoye**: Conceptualization (supporting); methodology (supporting); project administration (lead); supervision (lead); visualization (supporting); writing—original draft (supporting); writing—review and editing (equal). **Muritala Abiola Asafa**: Methodology (supporting); writing—review and editing (equal). **Kelechi Nelson Adindu**: Formal analysis (supporting); writing—review and editing (equal). **Omobolanle Suliyat Ahmed**: Writing—review and editing (equal). **Tomiwa Akande**: Writing—review and editing (equal).

## FUNDING INFORMATION

This research did not receive any specific grant from funding agencies in the public, commercial, or not‐for‐profit sectors.

## CONFLICT OF INTEREST STATEMENT

The authors declare that they have no conflicts of interest.

## DECLARATION OF GENERATIVE AI

No generative AI tools were used in the preparation/writing of this manuscript.

## Data Availability

The data that support the findings of this study are available from the corresponding author upon reasonable request.
